# Primary immunodeficiency diagnosed at autopsy: a case report

**DOI:** 10.1186/1756-0500-7-425

**Published:** 2014-07-04

**Authors:** Edwin Walong, Emily Rogena, David Sabai

**Affiliations:** 1 Anatomic Pathology Unit, Department of Human Pathology, School of Medicine, University of Nairobi, PO Box 19676, Nairobi, Kenya

**Keywords:** Primary immunodeficiency, DiGeorge syndrome, autopsy, Immunohistochemistry

## Abstract

**Background:**

DiGeorge syndrome may manifest as severe immunodeficiency diagnosed at infancy. The diagnosis of primary immunodeficiency is based on characteristic clinical features, immunophenotyping by flow cytometry, molecular diagnostics and functional lymphocyte evaluation. At autopsy, gross evaluation, conventional histology and immunohistochemistry may be useful for the diagnosis of primary immunodeficiency. This case report illustrates the application of autopsy and immunohistochemistry in the diagnosis of DiGeorge syndrome.

**Case presentation:**

A four-month-old African female infant died while undergoing treatment at Kenyatta National Hospital, a Referral and Teaching Hospital in Nairobi, Kenya. She presented with a month’s history of recurrent respiratory infections, a subsequent decline in the level of consciousness and succumbed to her illness within four days. Her two older siblings died following similar circumstances at ages 3 and 5 months respectively. Autopsy revealed thymic aplasia, bronchopneumonia and invasive brain infection by *Aspergillus species*. Microbial cultures of cerebrospinal fluid, jejunal contents, spleen and lung tissue revealed multi drug resistant *Klebsiella* spp, *Pseudomonas* spp, *Serratia* spp and *Escherichia coli*. Immunohistochemistry of splenic tissue obtained from autopsy confirmed reduction of T lymphocytes.

**Conclusion:**

Use of immunohistochemistry on histological sections of tissues derived from autopsy is a useful adjunct for post mortem diagnosis of DiGeorge syndrome.

## Background

DiGeorge syndrome is a congenital disorder characterised by third and fourth pharyngeal arch developmental anomalies, aetiology being micro-deletions of chromosome 22 q 11.2 [1]. Immunodeficiency may occur in DiGeorge syndrome manifested by quantitative and qualitative T lymphocyte deficits [2]. Severe infections particularly deep fungal infections are associated with a high morbidity and mortality in affected persons [3].

Among persons of African descent, DiGeorge syndrome presents with subtle craniofacial anomalies and is therefore underdiagnosed [4]. Furthermore, the diagnosis of primary immunodeficiency is limited by the high prevalence of paediatric infectious disease and low index of suspicion [5].This may explain the paucity of case reports on primary immunodeficiency due to DiGeorge syndrome in Africa.

Autopsy diagnosis of DiGeorge syndrome is important to identify index cases for subsequent genetic counselling, evaluate the pathogenesis and clinical features. We hypothesize that lymphocyte immunohistochemistry in lymphoid organs such as the spleen is a useful diagnostic adjunct for diagnosis of primary immunodeficiency. There are no reports on the utility of immunohistochemistry in diagnosis of primary immunodeficiency. We present a case of severe immunodeficiency due to suspected DiGeorge syndrome, whose diagnosis was aided by immunohistochemistry.

## Case presentation

The decedent was a four-month-old African female infant who presented with a five day history of fever, lethargy and refusal to feed. She was in a poor general condition, had peri-orbital oedema, cold extremities, restless with reduction in the level of consciousness. A blood gas analysis performed at admission revealed metabolic acidosis, while laboratory analysis of renal function test and liver function test revealed reduction in glomerular filtration rates and hyper-bilirubinemia respectively. She received broad spectrum antibacterial treatment and supportive care. Her condition worsened and died on the sixth day. On enquiry of the family history, it was revealed that the deceased two older siblings died of similar circumstances aged three and five months respectively. The autopsy was carried out on request from the family.

**Figure 1:**
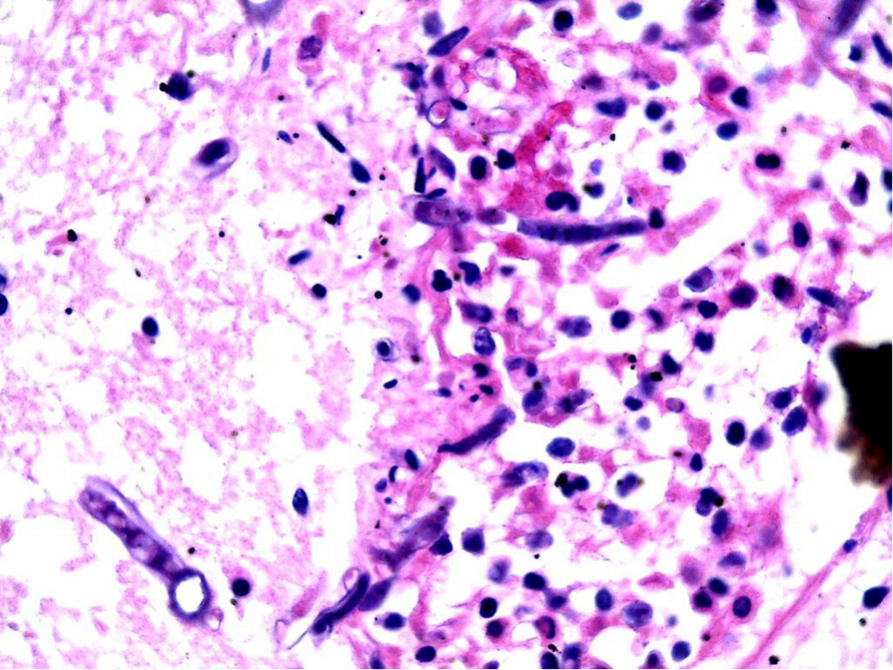
Photomicrograph of the brain abscess stained with Haematoxylin and Eosin, shows a mycotic brain abscess.

**Figure 2:**
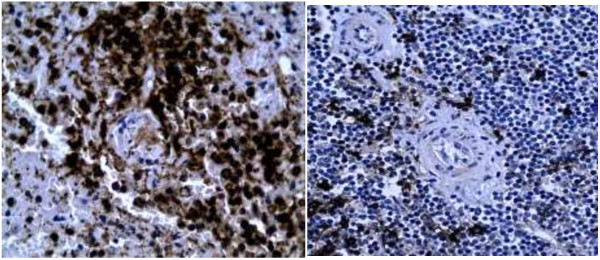
Photomicrograph of the spleen (case and age matched control), Immunohistochemistry for CD 20 (B lymphocytes).

**Figure 3:**
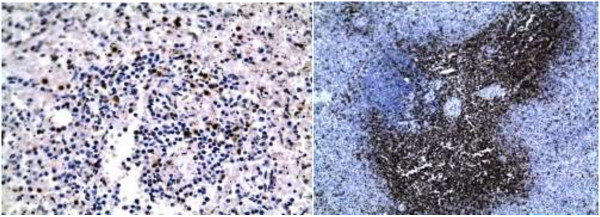
Photomicrograph of the spleen (case and age matched control), immunohistochemistry for CD 3 (T lymphocytes).

Autopsy was performed four days after death. The decedent was a well-developed infant whose appearance was consistent with the stated age of four months. On internal examination, the thymus was not grossly identified. There were frontal brain abscess with dark necrotic centres (Figure 1). The lungs showed evidence of bronchopneumonia. Microbiological culture in specimens obtained by aseptic techniques resulted in the isolation of multidrug resistant *Escherichia coli* in blood, cerebrospinal fluid and jejunal contents; *Serratia spp* in jejunal and blood cultures; *Proteus spp and Klebsiella spp* in cultures of jejunal contents, blood and splenic tissue*.* Histology of the lung showed diffuse alveolar damage, brain histology showed evidence of meningoencephalitis and mycotic abscess organisms consistent with *Mucor* or *Aspergillus spp* while liver histology showed clear cytoplasmic inclusions.Immunohistochemistry, CD 20 and CD3, was performed on splenic tissue to evaluate the distribution of lymphocytes within the white and red pulp. This showed B lymphocytes distributed within the peri-arteriolar lymphatic sheath (Figure 2). When compared to control splenic tissue from an age matched infant without immunodeficiency confirmed a marked reduction in T lymphocytes (Figure 3).

## Discussion

This case illustrates the diagnostic challenges of primary immunodeficiency and DiGeorge syndrome at autopsy. The incidence of DiGeorge syndrome in Africa is unknown [5]. Characteristic craniofacial features are not overtly detectable among persons of African descent bearing the genetic lesion associated with DiGeorge syndrome [4]. In this case, the absence of the thymus and severe immunodeficiency is highly suggestive of DiGeorge syndrome in contrast to velacardiofacial syndrome, which is characterised by cardiac and pharyngeal arch malformations [6].

Peripheral blood quantitative lymphocyte immunophenotyping by flow cytometry is useful for evaluation of qualitative and quantitative lymphocyte deficits for diagnosis of primary immunodeficiency [5]. We hypothesize that diagnostic lymphocyte defects can be demonstrated in primary lymphoid tissue specimens. This can be achieved by immunohistochemistry, which is widely available and accessible. Interpretation was based on comparisons to age matched controls in the appropriate clinical context.

Mycotic infections are a consistent feature of primary immunodeficiency syndromes characterised by defects in cellular immunity [3]. As revealed by the autopsy findings, the brain had abscesses with foci containing fungal hyphae whose morphology is consistent with *Aspergillus* spp. The presence of deep mycotic lesions in children should raise the index of suspicion for primary immunodeficiencies [3,5].

In this case, the index case and her siblings’ clinical features are suggestive of DiGeorge syndrome whereas the parents are asymptomatic. The mutation associated with DiGeorge syndrome, microdeletions in chromosome 22q11, are common, occurring at a rate of 1:4000 with the majority showing no morphological evidence of disease [7]. Clinical features are manifested as a result of loss of function mutations of T-box1 (tbx-1) enhancer region encoded in 22q11, required for neural crest and fibroblast migration [7,8]. Haploinsufficiency resulting from spontaneous mutation and autosomal dominant inheritance is associated with disease when gene expression is required early in morphogenesis [9]. However, evaluation of inheritance is limited because specific tbx-1 mutations and multifactorial associations that predict severe immunodeficiency have not been established [6]. Establishment of tbx-1 mutations in persons of African descent is indicated due to subtle morphological features in this group [4].

## Conclusion

In Africa, sporadic cases of primary immunodeficiency do exist whose diagnosis requires high indices of diagnosis due to the high prevalence of infectious disease. Autopsy diagnosis may identify index cases by clinical-pathological correlation. Immunohistochemistry performed on splenic tissue using panels of antibodies to B and T lymphocytes is useful for the classification of primary immunodeficiency.

## Consent

Written informed consent was obtained from the patient's parents for publication of this Case Report and any accompanying images. A copy of the written consent is available for review by the Editor-in-Chief of this journal.

## Competing interests

The authors declare no competing interests.

## Authors' contributions

DS analysed the clinical, demographic and family history relevant to this case. ER performed the autopsy and histological examination of the autopsy tissue. EW performed the autopsy, analyzed, interpreted and performed photomicrography. All authors read and approved the final manuscript.
